# Medical complications and management of atypical anorexia nervosa

**DOI:** 10.1186/s40337-022-00720-9

**Published:** 2022-12-16

**Authors:** Megen Vo, Neville Golden

**Affiliations:** Division of Adolescent Medicine, Department of Pediatrics, Stanford University School of Medicine, 750 Welch Road Suite 210, Palo Alto, CA 94304 USA

**Keywords:** Eating disorder, Atypical anorexia nervosa, Other specified feeding or eating disorder, Medical complications, Treatment

## Abstract

Atypical anorexia nervosa (AAN) is a new diagnosis in the 5th edition of the Diagnostic and Statistical Manual. Patients with AAN have been recognized to have similar, if not more severe, medical and psychological complications compared with patients with typical Anorexia Nervosa; yet studies on medical complications and optimal treatment of AAN are lacking. Here we review what is known regarding medical presentation and management of patients with AAN.

## Abstract (summary)

Atypical anorexia nervosa (AAN) is an eating disorder in which people who were previously higher or overweight experience body image disturbance and lose weight through unhealthy behaviors, resulting in weight loss, though patients may remain normal or above the normal weight range for age. In this article, we review the medical effects of AAN and how this unhealthy weight loss affects each organ system, and what is known about treatment.

## Background

Atypical Anorexia Nervosa (AAN), a new diagnosis in the 5th edition of the Diagnostic and Statistical Manual of Mental Disorders (DSM-5) and currently listed under the category of “Other Specified Feeding or Eating Disorders (OSFED),” describes those individuals who meet all criteria for anorexia nervosa (AN) except that despite significant weight loss, weight is in the normal or above normal range [1]. Some of these patients previously had weights above the 85th to 90th percentile for age. Like those with AN, patients with AAN have intense fear of gaining weight and engage in potentially dangerous weight control behaviors such as dietary restriction, fasting, excessive exercise, self-induced vomiting, and the use of laxatives, diuretics, diet pills, herbal remedies or complementary and alternative medications, to prevent weight gain.

Because current weight is in the normal range, the diagnosis of an eating disorder is often missed under the false assumption that people of normal weight cannot have an eating disorder. As a result, patients with AAN often present for treatment late in the course of their illness [2]. In addition, AAN is often perceived by medical providers to be less serious than AN. However, the medical complications in patients with AAN can be just as severe as those with classic AN and the degree of eating disorder psychopathology can be even more severe [3, 4].

The proportion of patients with a history of being in larger bodies presenting to specialized eating disorders programs has increased dramatically in recent years, often accounting for 25–45% of patients admitted to inpatient medical stabilization units [2–7]. In one tertiary care program, the number of such patients admitted to their inpatient service for medical instability increased fivefold over a six-year period [6].

While there are some differences in the demographic characteristics in those with AAN compared with those with AN, with a greater proportion of male and racial minorities in those with AAN [7], there are many similarities between the two conditions, leading many to question whether AAN is a different condition from AN, or whether it is on the spectrum of the same condition. “Low body weight” is what distinguishes AAN from AN, however there is no consensus about the weight cut-off to differentiate the two conditions. Some clinical researchers have used a weight ≥ 90% median body mass index (BMI) to indicate a normal or above normal weight, or a BMI of ≥ 19.5 kg/m^2^ for adult females and ≥ 20.2 kg/m^2^ for adult males [3, 8], while others have used a weight ≥ 85% median BMI for adolescents, or a BMI ≥ 18.5 kg/m^2^ for adult females and ≥ 19.5 kg/m^2^ for adult males [4, 9].

To date, studies of the medical complications of eating disorders have largely focused on AN, here we review the literature regarding medical complications of AAN.

### Medical complications of atypical anorexia nervosa

It is worth noting that the medical complications associated with AAN are similar, if not, in some cases, more severe than those associated with typical AN, such as hypophosphatemia (low serum phosphorus) as discussed in the sections below. The frequency of some medical complications such as menstrual disturbances and low bone mass may be lower in AAN than in AN. Studies, including those by Peebles et al. and Garber et al. note that severity of weight loss appears to be a significant predictor of medical instability [4, 10]. While patients with AAN may present with current weights in the normal range, by losing weight rapidly through restriction, self-induced vomiting, or other disordered behaviors, medical complications result. Table 1 summarizes the medical complications seen in AAN.

**Table 1:** Medical Complications of Atypical Anorexia Nervosa

Fluid and Electrolytes	Dehydration
	Hypokalemia
	Hyponatremia
	Hypophosphatemia
	Hypomagnesemia
Renal	Reduced eGFR
Cardiovascular	Bradycardia
	Hypotension
Gastrointestinal	Bloating and constipation
	Delayed gastric emptying
	Elevated liver enzymes
	Gallstones
	Gastro-esophageal reflux
	Esophagitis
	Barrett esophagus
	Bloody diarrhea
	Rectal prolapse
Endocrine	Low estrogen (girls) or low testosterone (boys)
	Primary or secondary amenorrhea (girls)
	Low T3 syndrome
Hematologic	Anemia
	Leukopenia
	Thrombocytopenia
Skeletal	Low bone mineral density
	Possible increased bone fragility
Neurologic	Syncope
	Seizures

### Fluids and electrolytes

Electrolyte abnormalities associated with malnutrition in the setting of eating disorders are well described, and include hypokalemia (low serum potassium), hyponatremia (low serum sodium), hypomagnesemia (low serum magnesium) and hypophosphatemia (low serum phosphorus). Garber et al. found that in patients with AAN, greater weight loss from premorbid weight and longer duration of weight loss was associated with lower serum phosphorus levels on inpatient admission; historically, low weight was thought to be the largest risk factor for hypophosphatemia, however, recent evidence points to the importance of assessing the rate and amount of weight loss, regardless of presenting weight [4, 10]. Electrolyte derangements may be the result of losses due to disordered behaviors such as diuretic or laxative abuse or self-induced vomiting, or due to electrolyte shifts in the setting of refeeding syndrome.

As with other physiologic instabilities due to malnutrition, refeeding syndrome can occur in patients with AAN, as well as in patients with typical AN. Refeeding syndrome refers to multi-organ failure associated with the rapid intracellular shift of electrolytes in response to a sudden increase in insulin release due to increased carbohydrate intake after a period of malnutrition. This increase in nutritional intake leads to an insulin surge, driving phosphorus intracellularly to increase production of adenosine-5-triphosphate (ATP). Extracellular hypophosphatemia, is the biochemical hallmark of refeeding syndrome, and may be responsible for many of the clinical manifestations of the syndrome [11]. Refeeding syndrome can cause potentially fatal shifts in fluids in electrolytes, with patients demonstrating hypophosphatemia, hypokalemia, and hypomagnesemia, as well as free water retention and may also demonstrate thiamine deficiency; symptoms include altered mental status, seizures, and may lead to death if untreated [12].

### Renal

Impaired renal function has been described in patients with AAN; a recent study by Downey et al. noted 33% of inpatients hospitalized for medical stabilization of malnutrition had reduced estimated glomerular filtration rate (eGFR) on admission, with no difference between those diagnosed with typical AN and AAN; more rapid weight loss was associated with impaired renal function in these patients [13]; eGFR was noted to improve with improved nutrition. Garber et al. noted that longer duration of weight loss was associated with lower serum creatinine in patients with AAN [4]. While studies on underlying causes of impaired renal function in AAN are lacking, proposed mechanisms include volume depletion leading to decreased renal perfusion, as well as impaired osmoregulation.

### Cardiovascular

Weight loss and malnutrition in AAN may lead to significant cardiovascular consequences, including bradycardia (low heart rate), hypotension (low blood pressure), orthostasis (abnormal changes in heart rate or blood pressure in response to positional changes), and electrocardiographic (EKG) abnormalities. Bradycardia in restrictive eating disorders is thought to be due a combination of inadequate energy intake and increased vagal tone. Studies [3, 9] have demonstrated similar rates of bradycardia in AAN compared with typical AN, which occurs as a hibernation response in the setting of inadequate caloric intake; in a study by Sawyer et al. 24% of adolescent females admitted with AAN had bradycardia and 43% had orthostasis on presentation [3].

### Neurologic

Neurologic effects of AAN include seizures, which can be the result of hyponatremia or other electrolyte disturbances or inadequate cerebral perfusion due to orthostasis or hypotension. Studies have demonstrated alterations in brain function in AAN, including in the recognition of non-verbal facial cues, similar to findings in AN [14, 15]. While studies of AN have demonstrated structural changes of the central nervous system on neuroimaging, such as reduced grey matter volume, such changes have not been found in patients with AAN to date [16]. One study of 22 adolescents with newly diagnosed AAN found no difference in grey matter volume between patients with AAN and healthy controls, whereas studies of patients with AN have demonstrated reduced grey matter in the parietal and temporal lobes, cingulum, and precuneus [15]. Another study of white matter microstructure showed no difference between patients with AAN compared with healthy controls [16–18]. Ghrelin, a peripherally produced, centrally active endogenous hormone, which normally stimulates food intake and reduces insulin secretion, has been proposed as a mediator of white matter changes in AAN as well as AN. One study by Breithapt et al. noted that elevated ghrelin in patients with AAN as well as typical AN was associated with white matter changes in late pubertal females [19].

### Gastrointestinal

In our clinical experience, patients with AAN often report gastrointestinal symptoms as a result of malnutrition and disordered eating behaviors. Malnutrition itself can lead to delayed gastric emptying and reduced intestinal motility, leading to early satiety, nausea, bloating, constipation and even involuntary emesis[20]. It should be noted that patients with AAN can have self-inducted emesis. Recurrent vomiting, whether voluntary or involuntary, can result in esophageal tears, gastrointestinal reflux, esophagitis and may lead to precancerous changes in the esophageal epithelium known as Barrett’s esophagus, though studies specifically among patients with AAN are lacking.

Elevations in hepatic transaminases may be seen in AAN due to hepatic autophagy or as consequences of refeeding syndrome. Up to 20% of patients with AAN had elevations of liver transaminases on admission in one study by Garber et al. [4], with the highest elevation below 100 IU/L for aspartate transaminase (AST). One study of patients with AN demonstrated a range of transaminase elevation during the course of inpatient hospitalization between 41 and 4607 IU/L for AST and between 46 and 3216 IU/L for alanine transaminase (ALT) [21].

### Endocrine

Significant derangements in hypothalamic-pituitary–gonadal (HPG) function due to malnutrition have been described in AAN. Malnutrition suppresses pulsatile release of gonadotropic releasing hormone leading to amenorrhea in females and suppression of testosterone in males in AAN [22]. Sawyer et al. noted in a study including 42 patients with AAN, 33% presented with secondary amenorrhea and an additional 7% of patients with AAN had missed 1 or 2 consecutive menstrual periods prior to presentation [3]. The frequency of menstrual disturbances is generally lower than in AN [3, 23, 24]. Estrogen and testosterone are important mediators of bone health in adolescence and HPG axis suppression may lead to bone demineralization, discussed further below.

### Hematologic

While literature on the hematologic manifestations specifically in AAN is lacking, anemia (low red blood cell count), thrombocytopenia (low platelet count), and leukopenia (low white blood cell count) are recognized consequences of inadequate nutritional intake due to gelatinous transformation of the bone marrow with resultant decrease in cell line production [25]. Inadequate dietary intake of iron, folate and B12 may also contribute to inadequate production of cell lines.

### Skeletal

Reduced bone mineral density has been described in AAN as a consequence of HPG axis suppression. Adolescence is a unique time of development for many reasons, not least of which because peak accrual of bone mass occurs during this time; disruption of the normal hormonal axis can lead to inadequate bone deposition, leading to reduced bone mineral density and osteoporosis in patients with AAN. Studies of dual x-ray absorptiometry data have demonstrated patients with AAN have higher bone mineral density than patients with typical AN but lower than healthy controls [23, 26, 27]. One study of bone mineral density in adolescents with AAN estimated a 34.2% lifetime risk of low bone mineral density [28]. In addition to the influence of estrogen and testosterone on osteoblast and osteoclast activity, other hormones including growth hormone (GH), insulin-like growth factor-1 (IGF-1, which stimulates bone growth and mineral deposition), ghrelin (which stimulates bone formation), and leptin (which stimulates bone resorption) play a role in normal bone deposition and can be disrupted by inadequate dietary intake and resultant hormonal suppression, leading to increased bone resorption as well as decreased deposition [29]. Data from patients with AAN are lacking, and the above derangements are extrapolated from AN literature.

### Medical management of atypical anorexia nervosa

Studies regarding optimal medical management of patients with AAN are few, and management of patients who were previously high weight poses unique challenges for the clinician. First and foremost, medical stabilization of vital sign abnormalities through nutritional rehabilitation, with careful monitoring of electrolytes, is key [30]. Determining the patient’s treatment goal weight should be individualized and based on the individual’s previous growth trajectory, which may translate to a treatment goal weight above the 50th percentile BMI for age. For adolescent patients who were previously above the 95th percentile BMI, we suggest using the patient’s vital signs and menstrual history (in individuals assigned female sex at birth) to guide treatment goal weight; if vital signs and menses stabilize before reaching the patient’s premorbid weight, additional weight gain may not be necessary [22]. Similarly, for patients without available growth records, we suggest using the clinical signs of recovery as above. One study of females with AAN showed that patients who were previously overweight did need to gain weight in order to resume menses, though slightly less than females with typical AN, 7.2 ± 5.9 kg compared with 8.9 ± 5.1 kg, *p* = 0.25[22]. Seetharaman et al. found that in females who were previously overweight, resumption of menses occurred on average at 106.1% ± 11.7% median BMI, compared to 94.2 ± 8.9 in those who were not previously overweight (*p* < 0.001) [31].

A multidisciplinary team, including a medical provider, registered dietitian, and therapist specializing in treatment of eating disorders, can provide the patient and family with comprehensive care during recovery. While early weight gain has been associated with increased rates of remission in AN [32], studies regarding optimal rate of weight gain in AAN are lacking. We suggest aiming for consistent weight gain until vital sign stability and resolution of amenorrhea are achieved, in addition to considering a return to the patient’s previous growth percentile, as discussed above.

The use of pharmacotherapy in AN and bulimia nervosa has been explored, however, evidence regarding the use of pharmacologic agents in AAN is lacking [20]. Selective serotonin reuptake inhibitors have not been shown to improve weight gain, depression or anxiety in patients with AN; it is hypothesized that inadequate nutritional intake impedes serotonin production, therefore less is available to reuptake even when SSRIs are used [33]. However, fluoxetine has been associated with improvement in binge eating and purging in patients with bulimia nervosa [34]. Therefore, patients with AAN who experience binge eating and purging may benefit from fluoxetine. Other medications, including atypical antipsychotics, have not been studied in AAN.

One case series of psychological treatment in adolescents with AAN suggest that family-based therapy can be effective in AAN, and can improve eating disorder symptoms [35]. While other therapeutic modalities have been studied in AN, there is a lack of data regarding the utility of other approaches for patients with AAN.

## Conclusions

The medical complications stemming from inadequate nutritional intake in AAN can affect many organ systems. Literature focused on medical complications specific to AAN is lacking and further study of the significant effects of weight loss among patients who were previously high or overweight is needed, especially in light of recent studies which demonstrate that the amount and duration of weight loss may be more predictive of electrolyte derangements than low weight alone [4]. Furthermore, studies regarding optimal treatment of patients with AAN are few, however, we suggest targeting return of menses and resolution of vital sign instability to guide determination of treatment goal weight, as well as taking into account the patient’s historical growth curves, which may necessitate a return to a BMI percentile above the 50th percentile. Utilizing the help of a multidisciplinary treatment team which may include a dietitian, therapist experienced in the treatment of eating disorders, in addition to the medical provider is recommended. Future studies should focus on the laboratory, particularly hormonal, markers of adequate nutritional status among patients with AAN, given the clinical conundrum of determining treatment goal weight in patients who have a history of being in larger bodies.

## Data Availability

Not applicable.

## Abbreviations

AAN: Atypical anorexia nervosa
AN: Anorexia nervosa
OSFED: Other specified feeding or eating disorder
DSM-5: Diagnostic and statistical manual, 5th edition
BMI: Body mass index
ATP: Adenosine-5-triphosphate
EKG: Electrocardiogram
eGFR: Estimated glomerular filtration rate
HPG: Hypothalamic pituitary gonadal
